# Double Aortic Arch Associated with Pulmonary Atresia with Ventricular Septal Defect

**DOI:** 10.5935/1678-9741.20160008

**Published:** 2016

**Authors:** Fernando Cesar Gimenes Barbosa Santos, Ulisses Alexandre Croti, Carlos Henrique De Marchi, Sírio Hassem Sobrinho

**Affiliations:** 1 Serviço de Cardiologia e Cirurgia Cardiovascular Pediátrica de São José do Rio Preto - Hospital da Criança e Maternidade de São José do Rio Preto and Faculdade de Medicina de São José do Rio Preto (FAMERP), São José do Rio Preto, SP, Brazil.

**Table:** 

**Abbreviations, acronyms & symbols**	
ICU	= Intensive care unit
PTFE	= Polytetrafluoroethylene
TGA	= Transposition of the great arteries
VSD	= Ventricular septal defect

## CLINICAL DATA

Preterm newborn at the 35^th^ week, second day of life, 2.7 kg, male, referred to our service after presenting respiratory distress associated with cyanosis. Upon physical examination presented at a regular general condition, eupneic in use of an oxygen mask with saturation around 97%. Presence of systolic murmur 4+/6+ predominantly at the lower left sternal border. Clear lung sounds. No abdomen findings. Present and symmetrical peripheral pulses.

## RADIOGRAPHY

Enlarged cardiac area mainly due to right atrial enlargement. Suggestive right aortic arch. Pleural-pulmonary spaces unchanged.

## ECHOCARDIOGRAM

Situs solitus in levocardia. Normal venoatrial and atrioventricular connections. Presence of wide perimembranous ventricular septal defect (VSD), with a bidirectional flow without significant gradient on Doppler. Confluent pulmonary arteries (diameter: trunk 4.7 mm/3.9 mm right pulmonary artery/3.6 mm left pulmonary artery), absent right ventriculoarterial connection. Right aortic arch in continuity with the descendent aorta originating the right common subclavian and carotid arteries. Left aortic arch originating the left common subclavian and carotid arteries, interrupted right after the emergence of the patent ductus arteriosus which have a diameter of 2.2 mm.

## COMPUTED TOMOGRAPHY ANGIOGRAPHY

**Fig. 1:**
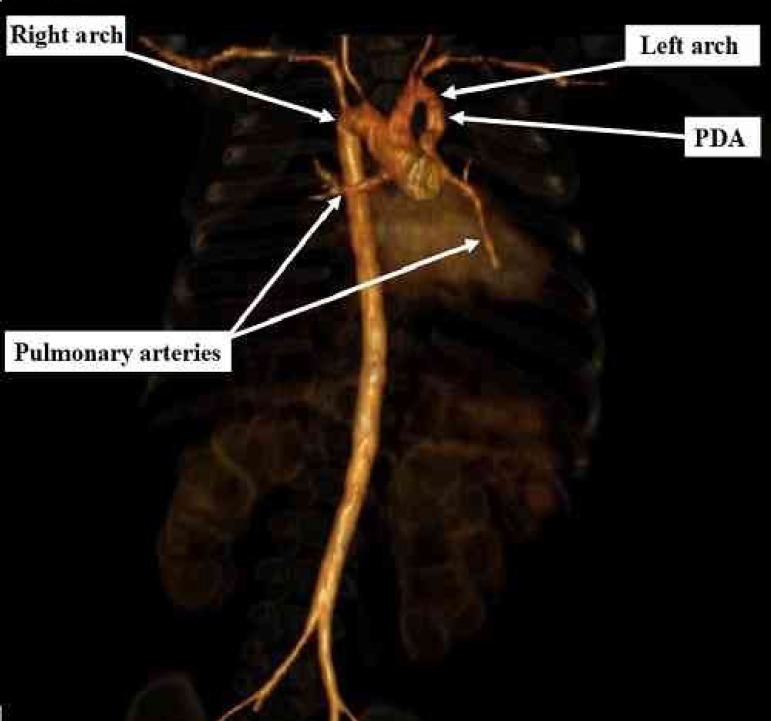
Three-dimensional reconstruction of computed tomography angiography showing both aortic arches, right and left. Note that the right arch is continuous with the descending aorta, and the left arch is interrupted right after the emergence of the ductus arteriosus which connects in the confluence of the right and left pulmonary arteries. PDA=patent ductus arteriosus

No typical image of pulmonary valve or trunk suggesting pulmonary atresia. Descendent aorta positioned to the right of midline. Double aortic arch without typical vascular ring formation. The dominant arch is positioned to the right with a diameter of 6.7 mm. The second arch is smaller (diameter of 4.9 mm), continued to the left as patent ductus arteriosus (average size of 3.7 mm) providing blood supply to the right and left confluent pulmonary arteries (Figure 1). There was also a perimembranous VSD of about 7 mm.

## DIAGNOSIS

It is known that the double aortic arch is the most common form of vascular ring and can be defined as a congenital anomaly in which the aortic arch and its branches surround the trachea and the esophagus completely or incompletely possibly causing compression of these structures^[1,2]^.

The first description of this disease were apparently done by Hommel, in 1737, and the first surgical correction was performed by Gross^[3]^, in 1945.

It should be thought of double aortic arch in patients with dysphasia, stridor, cough, dyspnea, and upper respiratory tract infections^[4]^. It can be diagnosed with the aid of echocardiography^[1,5,6]^, axial computed tomography^[6,7]^, magnetic resonance imaging^[6,8]^, contrast esophagogram^[4,6]^ and bronchoscopy^[4,6]^.

The most common form of double aortic arch is dominant right aortic arch, similar to the case presented, being present at 70% of the time. In 25% of cases there is a left dominant aortic arch and the remaining 5% can be two arches of the same size^[1]^.

Its correlation with other cardiovascular abnormalities, this malformation is less common. Backer et al.^[4]^ reported this correlation in only 26 (12.4%) in a series of 209 patients submitted to complete vascular ring surgical correction at Children's Memorial Hospital, in Chicago. When it occurs, it is often associated with a VSD although it can also occur with atrial septal defect, patent ductus arteriosus, tetralogy of Fallot or transposition of the great arteries (TGA)^[1]^.

Although the child may not present apparent cyanosis and clinical condition not drawing much attention, the echocardiogram was instrumental in the initial diagnosis and it demonstrated double aortic arch and pulmonary atresia with VSD. The additional anatomic details to orient the operation were obtained with the aid of the computed tomography angiography.

## OPERATION

Because it was a case of pulmonary atresia with VSD, there was a necessity to perform a systemic-pulmonary shunt, reason why median sternotomy was chosen. To help the choice of what aortic arch must be sectioned, during the preoperative monitoring arterial lines were put into both radial arteries. With this, we could test it temporarily by occluding the vessels and observing the curves in the monitor.

**Fig. 2:**
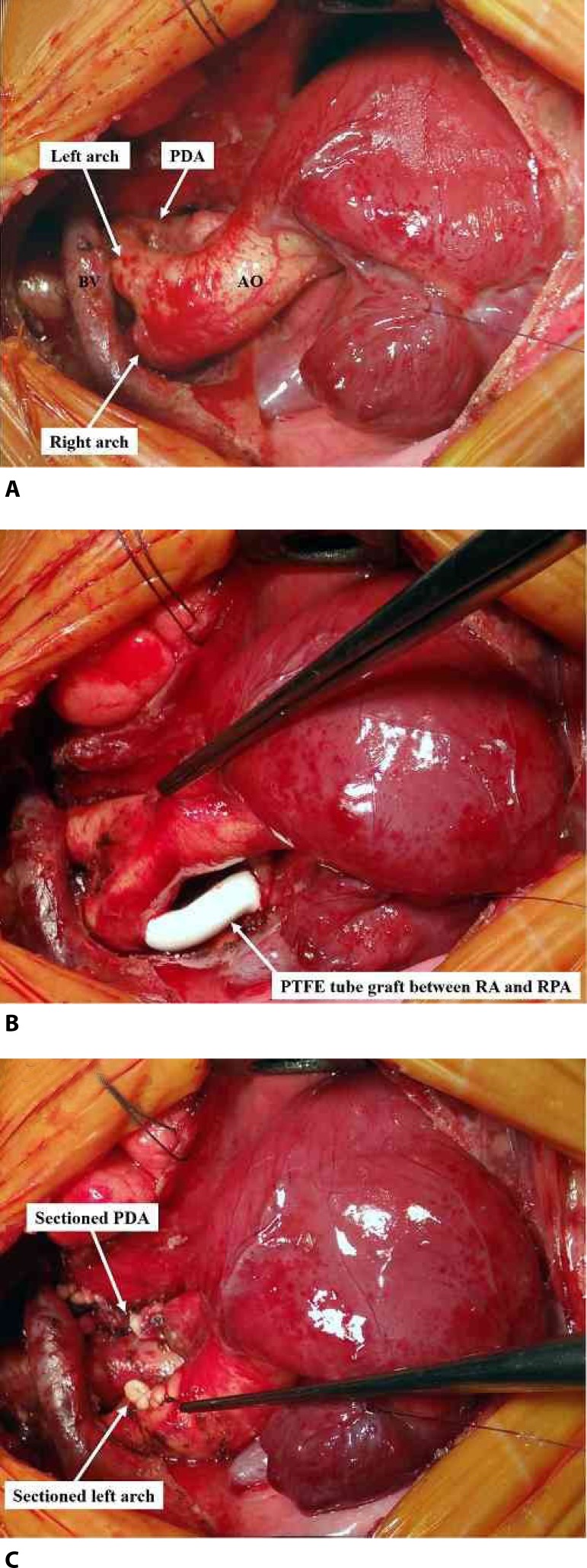
(A) Initial aspect of pulmonary atresia with ventricular septal defect and double aortic arch. The left aortic arch is incomplete and continues as patent ductus arteriosus that irrigates both pulmonary branches. The right aortic arch remains as descending aorta. (B) Modified Blalock-Taussig connecting the right aortic arch to the right pulmonary artery with 3.5 mm polytetrafluoroethylene (PTFE) tube. (C) Left aortic arch and ductus arteriosus sectioned and dried to remove extrinsic compression presumed after surgical evaluation. AO=aorta; BV=brachiocephalic vein; PDA=patent ductus arteriosus; RA=right arch; RPA=right pulmonary artery

After complete dissection of the aortic branches, the presence of both aortic arches and ductus arteriosus was evident (Figure 2A). After identifying the right aortic arch, a heparin dose weight of 2 mg/kg was administered and an interposition of the polytetrafluoroethylene (PTFE) of 3.5 mm connecting to the bottom side of the right aortic arch to the upper side of the right pulmonary artery (Modified Blalock-Taussig) with an 8-0 polypropylene suture was performed (Figure 2B). With effective pulmonary blood supply, which it was necessary since it was a ductus arteriosus dependent congenital heart defect, it was possible to section and suture it and immediately after, the left aortic arch, which was apparently compressing the esophagus, what wasn't seen in angiotomography (Figure 2C). The operation was performed normally without cardiopulmonary bypass.

After the procedure the patient had difficulty in weaning of the ventilator needing a tracheostomy and remained in the intensive care unit (ICU) for 20 days and have been in the semi intensive ICU for four months. He was discharged in excellent clinical conditions and in use of only aspirin.

**Table:** 

**Authors' roles & responsibilities**	
FCGBS	Manuscript writing and critical review of its content; final approval of the manuscript
UAC	Performed operations and/or experiments; manuscript writing or critical review of its content; final approval of the manuscript
CHM	Final approval of the manuscript
SHS	Final approval of the manuscript
